# Rate of gestational weight gain and preterm birth in relation to prepregnancy body mass indices and trimester: a follow-up study in China

**DOI:** 10.1186/s12978-016-0204-2

**Published:** 2016-08-12

**Authors:** Aiqun Huang, Zhenpeng Ji, Wei Zhao, Huanqing Hu, Qi Yang, Dafang Chen

**Affiliations:** 1 National Center for Women and Children’s Health, Chinese Center for Disease Control and Prevention, No.12, Dahuisi Road, Haidian District, Beijing, 100081 China; 2 Department of Epidemiology and Biostatistics, Peking University, School of Public Health, No.38, Xueyuan Road, Haidian District, Beijing, 100191 China

**Keywords:** Prepregnancy body mass indices, Rate of gestational weight gain, Preterm birth, Trimester, Chinese women

## Abstract

**Background:**

To evaluate the association between rate of gestational weight gain and preterm birth varying prepregnancy body mass indices and trimester.

**Methods:**

Data from Maternal and Newborn’s Health Monitoring System on 17475 pregnant women who delivered live singletons at ≥ 28 weeks of gestation between October 2013 and September 2014 from 12 districts/counties of 6 provinces in China and started prenatal care at ≤ 12 weeks of gestation was analyzed. Gestational weight gain was categorized by rate of weight gain during the 2^nd^ and 3^rd^ trimester, based on the 2009 Institute of Medicine guidelines. Multivariable binary logistic regression models were conducted to investigate the association between rate of gestational weight gain and preterm birth stratified by prepregnancy body mass indices and trimester.

**Results:**

Excessive weight gain occurred in 57.9 % pregnant women, and insufficient weight gain 12.5 %. Average rate of gestational weight gain in 2^nd^ and 3^rd^ trimester was independently associated with preterm birth (U-shaped), and the association varied by prepregnancy body mass indices and trimesters. In underweight women, excessive gestational weight gain was positively associated with preterm birth (OR 1.93, 95 % confidence interval (CI): 1.29- 2.88) when compared with women who gained adequately. While in overweight/obese women, insufficient gestational weight gain was positively associated with preterm birth (OR 3.92, 95 % CI: 1.13–13.67). When stratifying by trimester, we found that excessive weight gain in 3^rd^ trimester had a significantly positive effect on preterm birth (OR 1.27, 95 % CI: 1.02–1.58).

**Conclusions:**

Excessive gestational weight gain among underweight pregnant women, insufficient gestational weight gain among overweight/obese women and excessive gestational weight gain in 3^rd^ trimester were important predictors of preterm birth.

## Background

Preterm birth (PTB, <37 gestational weeks) has become an increasingly important global health concern, because of its association with infant mortality [1], long-term disability [2], and the high economic burden for health care it brings [3], particularly in developing countries [4]. Thus, identification of potentially modifiable risk factors to prevent PTB has become an important and meaningful work for researchers. In the past few decades, considerable studies on the determinants of PTB have been performed. An area of growing interest is the potential association between the maternal nutritional status before and during pregnancy and the occurrence of PTB.

Gestational weight gain (GWG), a strong indicator of pregnant woman’s nutritional status, is important for health and quality of life in women and their fetus. And many studies have been developed to investigate the relationship between maternal GWG and PTB. However, a recent study indicated that the conventional measurement of GWG--total GWG (kg), commonly used in previous epidemiologic studies, may bias the result [5]. Because of its inherent correlation with gestational age at delivery, we couldn’t figure out whether less total weight gain causes PTB, or just women who deliver at earlier gestation do not have enough time for weight gain compared to those who deliver at later gestational age. Thus, the measure of rate of weight gain (kg/week) in the 2^nd^ and 3^rd^ trimester, in which weight gain is relatively linear and constant, would be preferable when examining the relationship between maternal weight gain and PTB. In addition, 2009 Institute of Medicine (IOM) recommendations and two recent meta-analysis also acknowledged the dearth of research on the impact of GWG on PTB, particularly those with adjusted data and weekly GWG [6–8].

The maternal nutritional status before pregnancy, commonly measured through prepregnancy body mass indices (BMI), also has been reportedly associated with PTB [9–11]. While the association of PTB with underweight of prepregnancy has been recognized, whether prepregnancy overweight or obesity is associated with PTB is still undetermined [11–14]. Meanwhile, only few studies has examined the impact of prepregnancy BMI on the association between rate of GWG and PTB [15–17].

In addition, GWG used in previous epidemiologic studies were commonly the total amount, or GWG in specific trimester [16, 18, 19]. To our knowledge, no previous studies have simultaneously examined rate of GWG in 2^nd^/3^rd^ trimester in one population, to explore the impact of timing of GWG on PTB. Due to reasons above, in this study, we used the data from a monitoring system conducted in China to explore the influence of average rate of GWG in 2^nd^ and 3^rd^ trimester on PTB, particularly in different prepregnancy BMI groups and different trimesters.

## Methods

### Study site and population

Data for this analysis were obtained from Maternal and Newborn’s Health Monitoring System (MNHMS) by the National Center for Women and Children’s Health (NCWCH). The MNHMS was established to comprehensively monitor the prenatal health care and pregnancy outcomes information of pregnant women from 12 districts/counties of 6 provinces in 2013. The 6 provinces, including Hebei, Liaoning, Hunan, Fujian, Sichuan, Yunnan, then one city from each province and two districts/counties from each city were selected randomly by a three-stage cluster sampling. All pregnant women who were residents or lived more than 6 months at these places were enrolled at their first prenatal visit, and then their information before, during, and shortly after pregnancy would be collected in the system. The system contained information of large numbers of pregnant women, providing an opportunity to explore the associations between rate of GWG and PTB in a population-based sample.

### Data collection procedures

As mentioned above, all pregnant woman would be given perinatal health care cards at their first prenatal care visit and record data on the districts, type of residents, maternal race/ethnicity, maternal age, maternal education, and parity. Additionally, each pregnant woman received a complete physical examination including weight measurement, obstetric examination, hemoglobin examining, hepatic and renal function examination and ultrasound examination. The data of all subsequent prenatal care, which included weight measurement, obstetric examination and hemoglobin examining, were also collected in the perinatal health care cards by their doctors. Current pregnancy history including gestational age, delivery method, child’s sex, birth weight, Apgar score (1 min, 5 min), and other pregnancy outcomes were collected from their medical records during hospitalization. The perinatal health care cards would be collected when postpartum visiting at day 42 after childbirth and information on the cards would be input into the database of monitoring system.

### Data cleaning

We used the MNHMS database to obtain information for all pregnant women who delivered between October 2013 and September 2014 (*n* = 40679, 100 %). We excluded women with multiple pregnancies (*n* = 346, 0.85 %) to homogenize and simplify the calculations of rate of weight gain, as well as stillbirths (*n* = 70, 0.17 %). We further excluded women who delivered after 42 gestational weeks (*n* = 1050, 2.58 %), because we would use women who delivered between 37 and 42 weeks as reference group. We further selected women who started prenatal care before 13 weeks of pregnancy (*n* = 18117, 44.5 %), in order to approximately estimate prepregnancy weight using weight measurements in 1^st^ trimester because we did not have available information on the women’s prepregnancy weight measurements. Finally, we excluded women with implausible weight measurements and missing GWG data, leaving 17475 (42.96 %) women available for the analysis. When stratified by trimester, there were 497 and 587 pregnant women who had only one weight measurement and couldn’t calculate rate of weight gain in 2^nd^ and 3^rd^ trimester, leaving 16978 (97.2) and 16888 (96.6 %) analyzed, respectively.

### Maternal prepregnancy BMI and GWG

At enrollment and subsequent prenatal care, research assistants measured mother’s height (centimeters) and weight (kilograms) in light clothing without shoes. As we did not have information on the women’s prepregnancy weight measurements, maternal prepregnancy body mass index (BMI) was calculated using maternal weight and height at their first prenatal visit in 1st trimester (9.2 ± 2.3 gestational weeks). According to the IOM guidelines [8], we assumed that any pregnant women follow the normal pattern of weight gain in 1^st^ trimester: 2 kg average and increase linearly, calculating maternal prepregnancy BMI as: maternal weight at the first prenatal care visit *2/gestational week at the first prenatal care visit, like Margie H. Davenport’s study [20]. According to the World Health Organization’s Definition, prepregnancy BMI was categorized into four groups: underweight (BMI < 18.50), normal weight (18.50–24.99), overweight (25.00–29.99), and obese (BMI ≥ 30.00).

The measure of weight gain during pregnancy used in the study was average rate of GWG in 2^nd^ and 3^rd^ trimester, calculated by subtracting the women’s weight just prior to delivery (generally the week before delivery, but always less than 2 weeks before delivery) from her weight at the first prenatal care visit in the 2^nd^ trimester, and dividing this weight by the number of weeks between the two weight measurements. The second-trimester/third-trimester rate of GWG was calculated by dividing the difference of weight between the last and first prenatal care visit in the 2^nd^/3^rd^ trimester by the corresponding difference of gestational weeks, respectively. According to the 2009 Institute of Medicine (IOM) guidelines, rate of GWG was categorized as: insufficient, inadequate and excessive.

### Preterm birth

The main outcome of the study was the occurrence of PTB, defined as delivery occurring before 37 completed weeks of gestation. Gestational age can be obtained from pregnant women’s medical record, and was calculated by subtracting the last menstrual period from the date of delivery. Gestational age was categorized into PTB (<37 weeks of gestation),full term birth (≥37 and <42 weeks of gestation).

### Statistical analyses

Statistical analyses were performed with the SAS software (version 9.2; SAS Institute Inc., Cary, NC). Continuous variables were described using mean ± standard deviation and categorical variables were described using frequencies. Univariate logistic regression analysis was used to compare the maternal socio-demographic characteristics, pregnancy history, gestational weight gain between preterm birth and normal group (Table 1). Multinomial logistic regression was conducted to investigate the association between and PTB stratified by prepregnancy BMI and trimester and to control for the effect of confounders (Tables 2 and 3). Odds ratios (OR) and 95 % confidence intervals (CI) were calculated. To further explore the potential nonlinear relationship between GWG and PTB, we divided all women into ten groups according to rate of GWG (per 10 % a group), calculated adjusted ORs and 95 % CIs for PTB by GWG (group with lowest preterm birth rate as reference group) and plot the U shaped graph.

**Table 1:** PTB by different groups of characteristics, pregnancy history and GWG

Variables	N (%)	Mean (SD^a^)	OR (95 % CI^b^)	*P* value
Total	17475			
Districts				
Hebei	2871 (16.4)		reference	
Liaoning	1039 (5.9)		1.56 (1.12,2.16)	0.008
Xiamen	2080 (11.9)		0.64 (0.45,0.89)	0.009
Hunan	5294 (30.3)		0.82 (0.64,1.05)	0.119
Sichuan	1839 (10.5)		0.90 (0.66,1.24)	0.522
Yunnan	4352 (24.9)		0.99 (0.77,1.27)	0.911
Type of residents				
Local	16847 (96.4)		reference	
Non-local	628 (3.6)		0.49 (0.27,0.90)	0.021
Maternal race/ethnicity				
Han	16485 (94.3)		reference	
Other	990 (5.7)		1.50 (1.11,2.03)	0.008
Maternal education				
< =Junior high school	7060 (40.4)		reference	
Senior high school	3850 (22.0)		0.89 (0.72,1.11)	0.299
College or graduate school	3567 (20.4)		0.90 (0.73,1.13)	0.365
Missing	2998 (17.2)		0.81 (0.64,1.04)	0.093
Maternal age		26.7 (4.3)		
< 25	5633 (32.2)		reference	
25–35	10863 (62.2)		1.10 (0.92,1.32)	0.308
> = 35	979 (5.6)		1.89 (1.39,2.58)	<0.001
Maternal pre-pregnancy BMI (kg/m2)		20.7 (3.1)		
Underweight (<18.5)	4335 (24.8)		1.16 (0.96,2.00)	0.129
Normal (18.5–24.9)	11559 (66.1)		reference	
Overweight (25.0–30.0)	1347 (7.7)		1.30 (0.98,1.73)	0.072
Obese (> = 30.0)	234 (1.3)		1.48 (0.80,2.74)	0.210
Parity				
Nulliparous	10001 (62.2)		reference	
Multiparous	5098 (29.2)		1.04 (0.86,1.25)	0.696
Missing	2376 (13.6)		1.08 (0.85,1.37)	0.547
Infant sex				
Male	9241 (52.9)		reference	
Female	8234 (47.1)		0.87 (0.74,1.02)	0.094
Rates of weight gain (2nd and 3rd trimester) (kg/week)		0.56 (0.19)		
Insufficient	2298 (12.5)		1.46 (1.12,1.92)	0.006
Adequate	5054 (28.9)		reference	
Excessive	10123 (57.9)		1.38 (1.13,1.68)	0.002
Gestational age at delivery (weeks)		39.0 (1.3)		
Full-Term	16874 (96.6)			
Preterm	601 (3.4)			

^a^SD, Standard Deviation

^b^95 % CI, 95 % confidence interval

**Table 2:** Association between rate of GWG and PTB – stratified by pre-pregnancy BMI^a^

GWG rate (kg/week)		Preterm birth N (%)	Crude OR^c^ (95 % CI)	Adjusted OR (95 % CI)
Category^b^	Range			
Total (*N* = 17475)				
Insufficient	-	90 (3.9)	1.46 (1.12,1.92)	1.41 (1.07,1.85)
Adequate	-	137 (2.7)	reference	reference
Excessive	-	374 (3.7)	1.38 (1.13,1.68)	1.37 (1.11,1.68)
Underweight (*N* = 4335)				
Insufficient	<0.44	38 (4.0)	1.61 (1.01,2.56)	1.54 (0.97,2.47)
Adequate	0.44–0.58	36 (2.5)	reference	reference
Excessive	>0.58	87 (4.5)	1.81 (1.22,2.68)	1.93 (1.29,2.88)
Normal weight (*N* = 11559)				
Insufficient	<0.35	44 (3.5)	1.25 (0.87,1.80)	1.22 (0.85,1.76)
Adequate	0.35–0.5	97 (2.8)	reference	reference
Excessive	>0.5	232 (3.4)	1.22 (0.96,1.55)	1.20 (0.94,1.54)
Overweight/Obese (*N* = 1581)				
Insufficient	-	8 (9.5)	4.21 (1.23,14.42)	3.92 (1.13,13.67)
Adequate	-	4 (2.4)	reference	reference
Excessive	-	55 (6.0)	1.72 (0.62,4.81)	1.52 (0.53,4.32)

^a^Adjusted for maternal pre-pregnancy BMI, districts, type of residents, maternal race/ethnicity, maternal education, maternal age, parity and infant sex

^b^Based on the 2009 IOM Recommendations on weight gain during pregnancy

^c^OR, odds ratio

**Table 3:** Association between rate of GWG and PTB – stratified by trimester^a^

GWG rate (kg/week)	Preterm birth N (%)	Crude OR (95 % CI)	Adjusted OR (95 % CI)
Category^b^			
2nd trimester (*N* = 16978)			
Insufficient	119 (3.6)	1.16 (0.91,1.49)	1.11 (0.87,1.43)
Adequate	141 (3.1)	reference	reference
Excessive	330 (3.6)	1.16 (0.95,1.41)	1.11 (0.91,1.37)
3rd trimester (*N* = 16888)			
Insufficient	131 (3.2)	1.17 (0.91,1.51)	1.12 (0.87,1.44)
Adequate	117 (2.7)	reference	reference
Excessive	302 (3.6)	1.31 (1.05,1.63)	1.27 (1.02,1.58)

^a^Adjusted for maternal pre-pregnancy BMI, districts, type of residents, maternal race/ethnicity, maternal education, maternal age, parity and infant sex

^b^Based on the 2009 IOM Recommendations on weight gain during pregnancy

## Results

A total of 17475 women were included in the analysis. Table 1 shows the information on the maternal socio-demographic characteristics, pregnancy history, GWG, as well as the associations between PTB and these factors. Of these, 4335 women (24.8 %) were underweight, 11559 (66.1 %) had normal weights, 1347 (7.7 %) were overweight, and 234 (1.3 %) were obese. The mean rate of GWG in 2^nd^ and 3^rd^ trimester was 0.56 ± 0.19 kg/ week. According to the 2009 IOM recommendations, we categorized the weight gain into three categories: insufficient (2298, 12.5 %), adequate (5054, 28.9 %), and excessive weight gain (10123, 57.9 %). The mean rate of gestational age at delivery was 39.0 ± 1.3 week. Finally, 601(3.4 %) women had PTB. PTB was associated with the districts, type of residents, maternal race/ethnicity and maternal age. No differences were noted in occurrence of PTB at women in different group of education, parity and infant sex (Table 1).

As Table 2 shows, after adjustment for potential confounders, we found the average rate of GWG in 2^nd^ and 3^rd^ trimester was independently associated with PTB and the association was U-shaped: compared with women who gained adequately (reference), both low and high rates of weight gain could increase the risk of PTB: [OR 1.41 (95 % CI 1.07, 1.85)] for low rates of weight gain; [OR 1.37 (95 % CI 1.11,1.68)] for high rates of weight gain. And the stratification analysis showed that the association between PTB and the rate of GWG varied by the prepregnancy BMI: in women who were underweight, excessive GWG was positively associated with PTB [OR 1.93 (95 % CI 1.29,2.88)] when compared with women who gained adequately, while insufficient weight gain was not statistically significant; in women of normal weight, both excessive and insufficient GWG was not significantly associated with PTB; in women who were overweight/obese (for scarce number of obese group, we pooled data of the two groups), insufficient GWG was positively associated with PTB [OR 3.92 (95 % CI 1.13,13.67)] when compared with women who gained adequately, while excessive weight gain was not statistically significant.

We further conducted stratification analysis to explore the influence of weight gain in different trimester, and found that in 2^nd^ trimester both insufficient and excessive weight gain were positively associated with PTB when comparing with the reference group (adequate) but not statistically significant; While in 3^rd^ trimester only excessive weight gain were significantly associated with PTB [OR 1.27 (95 % CI 1.02,1.58)] when comparing with the reference group (adequate).

**Fig. 1:**
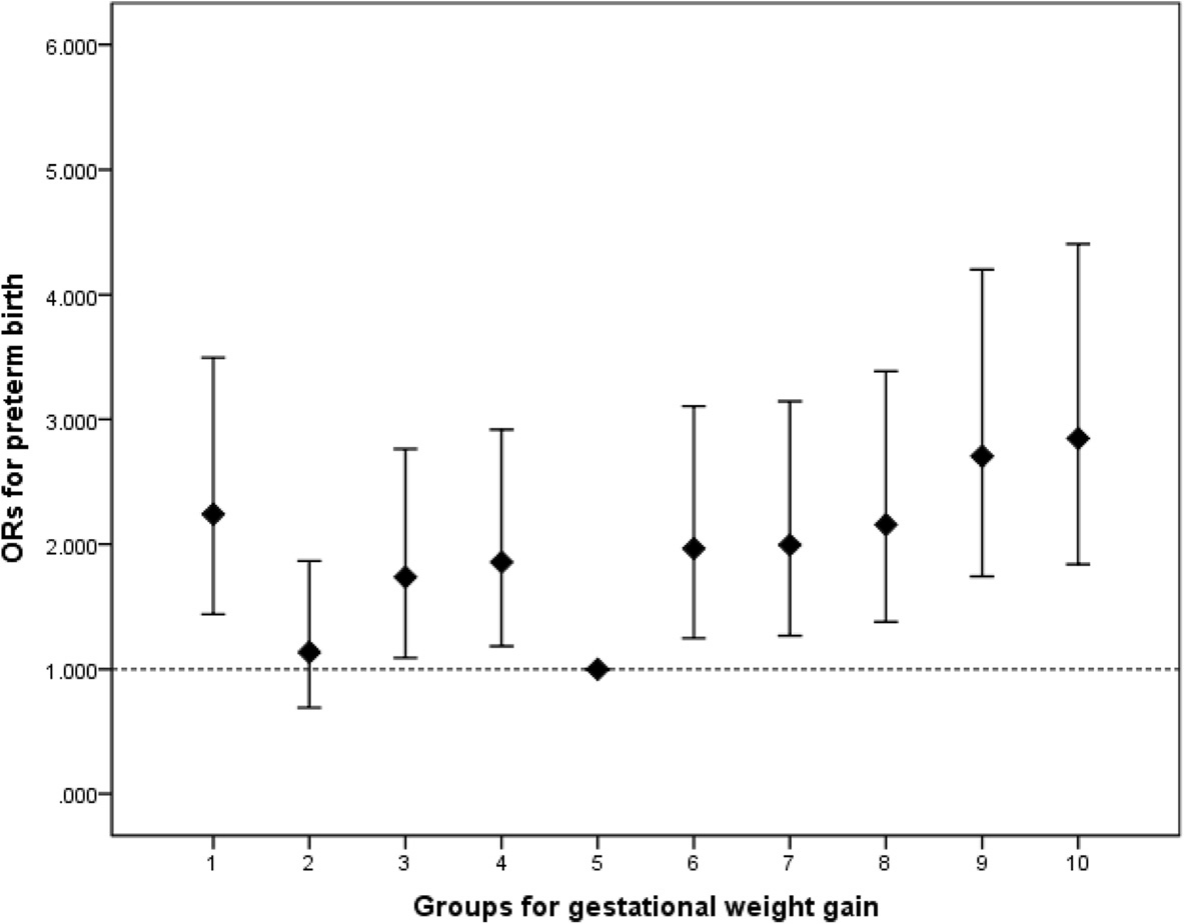
Adjusted ORs and 95 % CIs for preterm birth by gestational weight gain

We divided all women into ten groups according to GWG (per 10 % a group) to further explore the potential nonlinearity of the relationship between GWG and PTB. Compared with the reference group (5^th^ group), ORs for PTB by GWG in both higher and lower group were greater than 1. The graph also demonstrated that the effect of gestational weight gain on preterm birth was approximately U-shaped (Fig. 1).

## Discussion

In this follow-up study of Chinese pregnant women, we found 57.9 % women had excessive weight gain according to the 2009 IOM recommendations. The results demonstrated that both low and high rate of GWG in 2^nd^ and 3^rd^ trimester were positively associated with PTB. And the subsequent stratification analysis showed that this U-shaped association was explained mainly by excessive GWG among underweight women and insufficient GWG among overweight/obese women. When stratified by trimester, we found that excessive GWG in 3^rd^ trimester, not in 2^nd^ trimester was a strong predictor of PTB.

Previous studies reported that gaining less weight among obese women and gaining more among underweight women are protective of PTB [11, 15, 18, 21, 22]. The researchers considered that low weight gain may indicate deficiencies in micronutrients, poor expansion of plasma volume, and an increased risk of infection and/or inflammation, which are underlying causes of PTB, and excess weight gain can be explained by induction of a pro-inflammatory state or the fluid retention associated with pre-eclampsia/eclampsia preeclampsia [23–26]. Thus, gaining less weight among obese women and gaining more among underweight women could prevent those potential risk factors associated with PTB and decrease the risk of PTB. While our study found that lower than the recommended rate of GWG among overweight/obese women and higher rate among underweight women could increase the risk of PTB, demonstrating that though the 2009 IOM Recommendations encourage underweight women gain more and overweight ones less, it still should be within limits.

In stratification analysis, our study revealed that both insufficient and excessive weight gain in 2^nd^ trimester were not associated with PTB, which was consistent with Andrea J. Sharma’ study, while excessive GWG in 3^rd^ trimester could increase the risk of PTB.^19^ Though the biological mechanism about effect of timing of GWG on PTB is still unclear, it is reasonable to assume that timing of excessive GWG was important in predicting PTB and pregnant women should pay more attention to excessive weight gain in 3^rd^ trimester.

Our study has several strengths worth considering. First, using a relatively large sample size, in a geographic, ethnically and culturally varied population from China, to seek the association between PTB and rate of weight gain is one of the major strengths of this study. Second, rate of weight gain used in our study to adjust for the influence of length of pregnancy, is a preferable measurement compared to total weight gain, commonly used in previous studies. Finally, serial antenatal weight measurements collected over the course of pregnancy allowed the calculation of trimester-specific GWG, providing a unique opportunity to assess the impact of timing of weight gain on PTB.

Despite its strengths, there were some limitations that should be considered when interpreting its results. First, as the prepregnancy weight couldn’t be available on their medical records, we had to rely on maternal weight and height at their first prenatal visit in 1^st^ trimester to approximately calculate prepregnancy BMI, and assuming that any pregnant women follow the normal pattern of weight gain in 1^st^ trimester. Although we recognize that the prepregnancy weight may have still been inaccurate, it is reasonable to assume that such misclassification would probably be small. Second, to achieve the above study objective, we only selected women who had their first prenatal visit in 1^st^ trimester, which restricted the generalization of findings in this study to those with appropriate antenatal care. Third, for lacking information of PTB subtypes, we could not explore whether the association between PTB and rate of weight gain could be explained by its subtypes and further explore the etiology of PTB. The last, the history of preterm birth is a strong confounder which should be considered, but the monitoring project didn’t collected this information.

## Conclusion

In sum, this study in Chinese pregnant women demonstrated that average rate of GWG in 2^nd^ and 3^rd^ trimester exhibited an independent association with PTB, which varied by the prepregnancy BMI and trimesters. Higher rate of GWG among underweight pregnant women, lower rate among overweight/obese women and excessive weight gain in 3^rd^ trimester were important predictors of PTB. These findings highlight the need for further studies in Chinese women to confirm our results and suggest that nutrition interventions before conception and during pregnancy should be implemented to prevent the potential deleterious effects.

## Abbreviations

BMI, body mass indices; GWG, gestational weight gain; IOM, Institute of Medicine; PTB, preterm birth

## References

[1] Black RE, Cousens S, Johnson HL, Lawn JE, Rudan I, Bassani DG (2010). Global, regional, and national causes of child mortality in 2008: a systematic analysis. Lancet.

[2] Lumley J (2003). Defining the problem: the epidemiology of preterm birth. BJOG: An International Journal of Obstetrics and Gynaecology.

[3] Petrou S. The economic consequences of preterm birth during the first 10 years of life. BJOG : an international journal of obstetrics and gynaecology. 2005;112 Suppl 1:10–5. 10.1111/j.1471-0528.2005.00577.x 15715587

[4] Goldenberg RL, Culhane JF, Iams JD, Romero R (2008). Epidemiology and causes of preterm birth. Lancet.

[5] Hutcheon JA, Bodnar LM, Joseph KS, Abrams B, Simhan HN, Platt RW (2012). The bias in current measures of gestational weight gain. Paediatr Perinat Epidemiol.

[6] McDonald SD, Han Z, Mulla S, Lutsiv O, Lee T, Beyene J (2011). High gestational weight gain and the risk of preterm birth and low birth weight: A Systematic Review and Meta-Analysis. Journal of Obstet Gynaecol Canada.

[7] Han Z, Lutsiv O, Mulla S, Rosen A, Beyene J, McDonald SD (2011). Low gestational weight gain and the risk of preterm birth and low birth weight: a systematic review and meta-analyses. Acta Obstet Gynecol Scand.

[8] IOM (Institute of Medicine), National Research Council (2009). Weight Gain During Pregnancy: Reexamining the Guidelines.

[9] Xinxo S, Bimbashi A, ZKakarriqi E, Zaimi E (2013). Association between maternal nutritional status of prepregnancy, gestational weight gain and preterm birth. Materia Socio-medica.

[10] Huang A, Jin X, Liu X, Guo S (2015). A matched case-control study of preterm birth in one hospital in Beijing, China. Reprod Health.

[11] Hendler I, Goldenberg RL, Mercer BM, Iams JD, Meis PJ, Moawad AH (2005). The Preterm Prediction Study: association between maternal body mass index and spontaneous and indicated preterm birth. Am J Obstet Gynecol.

[12] Ronnenberg AG, Wang X, Xing H, Chen C, Chen D, Guang W (2003). Low preconception body mass index is associated with birth outcome in a prospective cohort of Chinese women. J Nutr.

[13] McDonald SD, Han Z, Mulla S, Beyene J, Knowledge Synthesis Group (2010). Overweight and obesity in mothers and risk of preterm birth and low birth weight infants: systematic review and meta-analyses. Br Med J.

[14] Bhattacharya S, Campbell DM, Liston WA, Bhattacharya S (2007). Effect of Body Mass Index on pregnancy outcomes in nulliparous women delivering singleton babies. BMC Public Health.

[15] Fujiwara K, Aoki S, Kurasawa K, Okuda M, Takahashi T, Hirahara F (2014). Associations of maternal prepregnancy underweight with small-for-gestational-age and spontaneous preterm birth, and optimal gestational weight gain in Japanese women. J Obstet Gynaecol Res.

[16] Carnero AM, Mejia CR, Garcia PJ (2012). Rate of gestational weight gain, prepregnancy body mass index and preterm birth subtypes: a retrospective cohort study from Peru. BJOG : an international journal of obstetrics and gynaecology.

[17] Schieve LA, Cogswell ME, Scanlon KS (1999). Maternal weight gain and preterm delivery: differential effect by body mass index. Epidemiology.

[18] Spinillo A, Capuzzo E, Piazzi G, Ferrari A, Morales V, Di Mario M (1998). Risk for spontaneous preterm delivery by combined body mass index and gestational weight gain patterns. Acta Obstet Gynecol Scand.

[19] Sharma AJ, Vesco KK, Bulkley J, Callaghan WM, Bruce FC, Staab J (2015). Associations of gestational weight gain with preterm birth among Underweight and Normal Weight Women. Matern Child Health J.

[20] Davenport MH, Ruchat SM, Giroux I, Sopper MM, Mottola MF (2013). Timing of excessive pregnancy-related weight gain and offspring adiposity at birth. Obstet Gynecol.

[21] Dietz PM, Callaghan WM, Cogswell ME, Morrow B, Ferre C, Schieve LA (2006). Combined effects of prepregnancy body mass index and weight gain during pregnancy on the risk of preterm delivery. Epidemiology.

[22] Schieve LA, Cogswell ME, Scanlon KS, Perry G, Ferre C, Blackmore-Prince C (2000). Prepregnancy body mass index and pregnancy weight gain: associations with preterm delivery. Obstet Gynecol.

[23] Carmichael SL, Abrams B (1997). a critical review of the relationship between gestational weight gain and preterm delivery. Obstet Gynecol.

[24] Wen SW, Goldenberg RL, Cutter GR, Hoffman HJ, Cliver SP (1990). Intrauterine growth retardation and preterm delivery: prenatal risk factors in an indigent population. Am J Obstet Gynecol.

[25] Siega-Riz AM, Promislow JH, Savitz DA, Thorp JM, McDonald T (2003). Vitamin C intake and the risk of preterm delivery. Am J Obstet Gynecol.

[26] Barinas-Mitchell E, Cushman M, Meilahn EN, Tracy RP, Kuller LH (2001). Serum levels of C-reactive protein are associated with obesity, weight gain, and hormone replacement therapy in healthy postmenopausal women. Am J Epidemiol.

